# Infantile idiopathic scoliosis: surgical treatment in rapidly progressive cases

**DOI:** 10.1186/1748-7161-7-S1-P7

**Published:** 2012-01-27

**Authors:** D Lawniczak, T Kotwicki

**Affiliations:** 1 Spine Disorders Unit, Department of Pediatric Orthopedics and Traumatology, University of Medical Sciences, Poznan, Poland

## Purpose

To present 4 cases (5 curves) of infantile scoliosis in children initially managed with corrective brace and physiotherapy. All finally underwent surgical treatment.

## Materilas and methods

Age of onset was in between 4th and 36th month of life. Initial treatment consisted of physiotherapy and Chenau corrective bracing in all patients. Duration of conservative treatment was from 4 to 5.5 years. The age at surgery was: from 7 to 12 years (table 1).

**Table 1:** 

Patient	Age at diagnosis in years	Curve magnitude before treatment Cobb angle	Brace treatment since	Curve magnitude before surgery Cobb angle	Age at surgery	Curve magnitude at last follow up Cobb angle
1.	3	65	5	98	9	55
2.	3	48	6	78	10	53
3.Thoracic curve	0.5	47	1.5	65	7	40
3.Lumbar curve	0.5	44	1.5	56	7	54
4.	2	55	10	97	12	58

Finally patients underwent surgical treatment: anterior and posterior fusion in 2 patients (3 curves), posterior instrumentation (2 growing rods) in one patient, and VEPTR device in one case. All have led to clinical improvement.

## Results

In those cases the curve was rapidly progressing despite our efforts to stop it. As the conservative measures failed, we proceed to surgery for correction and stabilisation of the spine. There were no major complications during surgical treatment.

## Conclusions

Conservative treatment plays a vital role in treatment of scoliosis. However, in cases of early onset and rapid progression, surgical treatment appears to be a reliable method.

